# Managing a patient with bipolar disorder associated with COVID‐19: A case report from Qatar

**DOI:** 10.1002/ccr3.4015

**Published:** 2021-03-11

**Authors:** Mohamad Y. Khatib, Omer B. Mahgoub, Marwa Elzain, Amna A. Ahmed, Ahmed S. Mohamed, Abdulqadir J. Nashwan

**Affiliations:** ^1^ Critical Care Medicine Department Hazm Mebaireek General Hospital (HMGH) Hamad Medical Corporation (HMC) Doha Qatar; ^2^ Mental Health Services (MHS) Hamad Medical Corporation (HMC) Doha Qatar; ^3^ University of Calgary in Qatar Doha Qatar

**Keywords:** bipolar, COVID‐19, mental health, SARS‐CoV‐2

## Abstract

This case highlights an atypical presentation of a patient with unknown history of mental disease who has been diagnosed with a bipolar disorder associated with severe COVID‐19 symptoms. Neuroimaging was only positive for subtle white matter changes; he was treated with antipsychotics and mood‐stabilizing agents until he reached partial remission. The authors urge clinicians to consider the impact of the COVID‐19 pandemic on patients with mental illness and the urgent need for vigilant monitoring of presenting signs and symptoms.

## Graphical abstract

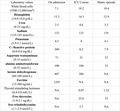

## 1 INTRODUCTION

The novel coronavirus disease 2019 (COVID‐19) is caused by the severe respiratory syndrome coronavirus 2 (SARS‐COV‐2) and according to the World Health Organization (WHO) declared the COVID‐19 outbreak a pandemic on 11th of March 2020, which require national and global measurement to control this pandemic and strong public health response.^1^ The presentation of COVID‐19 in most of the cases was in the form of cough, fever, sore throat, dyspnea and myalgia. In addition, the nervous system involvement, can be presented in the form of cerebrovascular disease,^2^ new‐onset anosmia, encephalitis and dysgeusia.^3^, ^4^ It has emerged in the COVID‐19 literature many cases of reactive psychosis, however, less attention has been given to incident psychosis in patients with COVID‐19.^5^ Here, we reported a case of new‐onset psychosis in a patient with no previous background of mental illness following a severe course of SARS‐COV‐2 infection.

## 2 CASE PRESENTATION

A 52‐year‐old man with a background of longstanding well‐controlled epilepsy. He was not known to have a previous history of any mental illness. He tested positive for the novel SARS‐COV‐2 virus on a nasopharyngeal and throat swab after community exposure to a confirmed case. He was asymptomatic at the time of testing positive but was admitted to a governmental quarantine facility as per the country's guidelines (Table 1). Eight days following his initial presentation, he was transferred from the quarantine facility to a designated COVID‐19 hospital after developing fever, cough, and shortness of breath. Clinical imaging confirmed the diagnosis of severe covid‐19 pneumonia; he was commenced on antibiotics (Oral azithromycin and intravenous cefuroxime), hydroxychloroquine, dexamethasone and oxygen therapy. Hemoglobin A1c was found to be high and he was diagnosed with diabetes mellitus and started on insulin. One week following hospitalization, he was noted to be confused and complained of severe headache and abdominal pain. He was transferred to the intensive care unit to be investigated and managed for an impression of an acute confusional state. The initial workup revealed hyponatremia for which he was started on hypertonic saline. Computed tomography of the brain did not show any abnormalities.

**TABLE 1:** laboratory investigations

Laboratory values	On admission	ICU Course	Manic episode
White blood cells (3500‐12 000/mm^3^)	7.1	18.2	10.4
Hemoglobin (14.0‐18.0 g/dL)	13.2	14.1	12.9
Urea (8‐23 mg/dL)	2.5	4.6	3.3
Sodium (135‐145 mmol/L)	133	123	135
Potassium (3.5‐5.1 mmol/L)	4.5	4	4.1
C‐ Reactive protein (0.0‐0.8 mg/dL)	309	8.2	7.9
Aspartate transaminase (0‐35 units/L)	71	31	23
alanine aminotransferase (0‐35 units/L)	100	104	33
lactate dehydrogenase (60‐100 units/L)	407	284	NA
Ferritin (15‐200 ng/mL)	1293	NA	NA
Thyroid stimulating hormone (0.4‐4.8 mIU/L)	NA	0.07	1.32
Free thyroxine (1.0‐2.1 ng/dL)	NA	25.6	15
free triiodothyronine (1.1‐2.9 nmol/L)	NA	3.7	NA

During his stay in the intensive care unit, he was assessed by the consultation‐liaison psychiatry team. The team's assessment was consistent with 5 days of an acute confusional state based on disorientation to time, impaired concentration and memory, visual and auditory hallucinations (seeing and hearing Jesus), persecutory beliefs against hospital staff, labile mood, and delinquent behavior requiring sedation with oral quetiapine and injectable haloperidol.

The patient responded well to treatment, he was transferred to a medical ward after spending four days in the intensive care unit and was discharged home two days later. Upon discharge, he was fully oriented, and his mental state examination did not show any abnormalities. He was discharged from the hospital on oral hypoglycemic agents, antiepileptic medications, and quetiapine 50 mg twice daily. His repeated Nasopharyngeal and throat swab was still Positive for SARS‐COV2 but was declared to be noninfectious based on an RdRp‐gene ct value of 32.3.

Two days after his discharge from the hospital, he presented to the emergency department following an episode of physical aggression in a hotel where he was residing, during the assessment, he justified his behavior by dereliction from the hotel staff. He was threatening to be physically aggressive if his demands were not immediately met. He was noted with a grandiose attitude and claimed to be a Master of Yoga. He demanded to be released from the hospital citing that he does not believe that he has any mental illness based on his medical, educational background. His family members and friends have reported that he was calling and messaging them repeatedly throughout the day.

His mental state examination was remarkable for psychomotor agitation, overfamiliar attitude, pressured speech, “very happy” mood, an expansive effect, persecutory and grandiose delusions and poor insight. Attention and orientation were found to be intact.

Physical and neurological examinations did not reveal any abnormalities.

His medical history is significant of epilepsy since childhood, he was well maintained on medications (levetiracetam 1000 mg and sodium valproate 1500 mg), his last seizure was 20 years prior to presentation. He was newly diagnosed with diabetes mellitus and was on two oral hypoglycemic agents. He denied any background of mental illness in himself or his family.

He had maintained a job as a financial consultant for the past 10 years. He is married for 25 years with 3 children. He was living alone, and his family resided in his home country. He is a nonsmoker and had no history of alcohol or illicit substance use.

## 3 INVESTIGATIONS

MRI of the brain showed subtle white matter changes which were ruled to be of no clinical correlation.

## 4 DIFFERENTIAL DIAGNOSIS

Bipolar affective disorder, manic episode with psychotic features.

Acute confusional state.

Mood disorder secondary to a general medical condition.

## 5 TREATMENT

He was started on risperidone at 1 mg once daily as an antipsychotic and mood‐stabilizing agent under the impression of a manic episode and a decision was made to admit him to an inpatient psychiatry ward for further management.

Risperidone was gradually increased up to a dose of 2 mg twice daily but was later reduced to 2 mg daily due to the emergence of bilateral hand tremors.

Clonazepam 1 mg at bedtime was added for a few days during his hospitalization as he was noted to have disturbed sleep.

His antiepileptic and oral hypoglycemic medications were resumed.

## 6 OUTCOME AND FOLLOW‐UP

He showed gradual improvement in his manic and psychotic symptoms over the course of his hospitalization and he has discharged home after 3 weeks on Risperidone 2 mg once daily. He was contacted via phone 2 weeks following discharge and he reported maintained improvement other than difficulties with the initiation of sleep for which his dose of Risperidone was increased to 3 mg once daily.

## 7 DISCUSSION

In this case, we present a middle‐aged patient with no previous mental illness background who presented with full‐blown manic symptoms following a severe course of SARS‐COV2 infection. There were few other reported cases of first onset manic‐like symptoms in patients following a severe SARS‐COV2 infection.^6^, ^7^ These cases discussed the possibility of the neuroinvasive properties of the virus being directly related to the emergence of symptoms given previous reports of CNS infections associated with the novel virus.^8^ However, they have highlighted the limitations of the lack of CSF‐PCR assays for validation of this hypothesis. Given the patient's age, lack of past personal or family psychiatric history and the prodrome of an acute confusional state preceding the onset of manic symptoms, a neuroinvasive etiology is possible. However, the possibility of a primary mood disorder cannot be dismissed without a longer period of follow‐up.

COVID‐19’s clinical manifestations range from no symptoms to a more severe form of the disease resulting in multiorgan failure and sepsis requiring ICU care and mechanical ventilation.^9^ Nalleballe et al reported neuropsychiatric manifestations in 22.5% of more than 40,000 patients who had COVID‐19; including Mood disorders, anxiety, stroke, seizures, and encephalopathy. The prevalence of mood disorders was estimated to be 3.8% in the study sample.^10^

The novel SARS‐COV2 virus neurotropic properties were discussed in the literature. Several mechanisms were proposed to explain the neuropsychiatric manifestation of COVID‐19 including direct cytokine network dysregulation, Central nervous system (CNS) infiltration, peripheral immune cell transmigration, and postinfectious autoimmunity.^11^

The possibility of a primary bipolar affective disorder cannot be ruled out in our patient. He initially experienced an acute confusional state during the acute phase of COVID‐19 infection as evident by disorientation and visual hallucinations which subsided before his discharge from the COVID‐19 facility. He later presented with classical features of mania after he was declared to have recovered from COVID‐19 and was in clear sensorium at that time. The patient's medical history of epilepsy can be considered another risk factor for developing a mood disorder. Reports from the United States revealed that around 12% of individuals with epilepsy screened positive for symptoms of bipolar disorder in comparison to 1 to 2% lifetime incidence in the general population.^12^

## 8 CONCLUSION

This case highlights the importance of giving attention to psychiatric symptoms in patient with COVID‐19 and early intervention and involvement of psychiatrist especially in critically ill patients. Though they may be no history of mental health or family history of psychiatric illness, the COVID‐19 may be associated with bipolar incidence especially in critically ill patients. A timely diagnosis and early psychiatric intervention can improve the outcome of patient. In the present scenario, we urge physicians to pay attention for those cases and reported them, so more studies can be done to identify the relation between COVID‐19 and psychiatric illness.

## 9 ETHICS APPROVAL AND CONSENT TO PARTICIPATE

The article describes a case report. Therefore, no additional permission from our Ethics Committee was required.

## 10 CONSENT FOR PUBLICATION

The consent for publication was obtained.

## CONFLICT OF INTEREST

The authors declare that they have no competing interests.

## AUTHOR CONTRIBUTION

MYK, OBM, MEE, AAA, ASM, AJN: Data Collection, Literature Search, Manuscript Preparation. All authors read and approved the final manuscript.

## Data Availability

All data generated or analyzed during this study are included in this published article.
